# Matching intraoperative teaching and learning for medical undergraduates via modified briefing-intraoperative teaching-debriefing (BID) model

**DOI:** 10.1038/s41598-023-40755-9

**Published:** 2023-08-23

**Authors:** Yu-Tang Chang, Nan-Chieh Chen, Shu-Hung Huang, Chung-Sheng Lai, Cheng-Sheng Chen, Ting-Wei Chang, Po-Chih Chang

**Affiliations:** 1 Division of Pediatric Surgery, Department of Surgery, Kaohsiung Medical University Hospital/Kaohsiung Medical University, Kaohsiung City, Taiwan; 2 School of Medicine, College of Medicine, Kaohsiung Medical University, Kaohsiung City, Taiwan; 3 Department of Medical Humanities and Education, College of Medicine, Kaohsiung Medical University, Kaohsiung City, Taiwan; 4 Division of Plastic Surgery, Department of Surgery, Kaohsiung Medical University Hospital/Kaohsiung Medical University, Kaohsiung City, Taiwan; 5 Division of Plastic Surgery, Department of Surgery, Kaohsiung Municipal Siaogang Hospital/Kaohsiung Medical University, Kaohsiung City, Taiwan; 6 Department of Psychiatry, Kaohsiung Medical University Hospital/Kaohsiung Medical University, Kaohsiung City, Taiwan; 7 Division of Thoracic Surgery, Department of Surgery, Kaohsiung Medical University Hospital/Kaohsiung Medical University, No. 100, Tzyou 1st Road, Kaohsiung City, 80756 Taiwan; 8 Weight Management Center, Kaohsiung Medical University Hospital/Kaohsiung Medical University, Kaohsiung City, Taiwan; 9 College of Medicine, Ph. D. Program in Biomedical Engineering, Kaohsiung Medical University, Kaohsiung City, Taiwan; 10 Department of Sports Medicine, College of Medicine, Kaohsiung Medical University, Kaohsiung City, Taiwan

**Keywords:** Health care, Health occupations, Medical research

## Abstract

Intraoperative teaching is a challenging task. The briefing–intraoperative teaching–debriefing (BID) model, which is based on guided discovery learning at limited time intervals, has rarely been investigated. This study validated the benefits of the modified BID model on medical clerks. This study involved 37 first-year medical clerks enrolled from September 2019 to May 2020. Every learner scrubbed in one the totally implantable venous access device placement surgery and completed a pre-/posttest survey on surgical procedures and associated anatomy conducted through an intraoperative teaching questionnaire. Of these participants, 15 merely observed throughout the entire procedure (observation group), whereas the remaining 22 performed simple suturing under supervision (suturing group). All participants underwent an objective structured assessment of simple interrupted suturing skills at the end of the observership. Correlations were tested using a two‐tailed paired t-test, with a *p*-value < 0.05 indicating statistical significance. The response rate was 100% and participants could reconfirm the precise venous access, catheter tip location, and suture materials for portal fixation after totally implantable venous access device placement (*p* < 0.05). Although a relatively higher satisfaction of the intraoperative teaching environment and educator attitude was reported in the suturing group than in the observation group, the difference in scores on the objective structured assessment was not statistically significant (8.7 ± 1.8 vs. 7.2 ± 3.7; *p* = 0.104). Our findings indicate that the modified BID model with hands-on experience is a practicable module for matching intraoperative teaching and learning via learning perception enhancement for medical undergraduates during totally implantable venous access device placement.

## Introduction

Intraoperative teaching is a challenge for surgical educators and learners. For decades, the training of junior surgical residents has been mostly based on apprenticeship^1,2^. Matching teaching and learning for junior learners (e.g., medical undergraduates) can be a clinically significant issue because of existing disparities and even contradictions^3^. However, few reports have been published on intraoperative teaching for medical undergraduates, which are currently accomplished through clinical observership and hands-on practice under direct supervision. Given the concern on fatigue and poor performance, surgical observership is less effective, even frustrating, for both medical undergraduates and surgical educators, which may render learners to become passive and less focused^4^. Thus, inspiring the interest of medical undergraduates has become fundamental to achieving effective intraoperative teaching in modern medical education.

Aside from traditional observership, the briefing–intraoperative teaching–debriefing (BID) model is sometimes adopted as a structured framework of intraoperative teaching for educators and learners^5–8^. The original concept of the BID model was proposed by Roberts et al. in 2009 and included three major subcomponents, namely, setting learning objectives, teaching during encounter, and reflection with reinforcement. The BID model not only emphasizes intraoperative teaching but also allows the learners to set learning objectives to meet their own needs and assemble their thoughts during the limited timeframe of that encounter^5^. Therefore, intraoperative teaching could be efficaciously established via the BID model for surgical educators and learners, even in busy clinical settings and operating rooms. Moreover, it could be a practicable intraoperative teaching solution for less advanced learners (e.g., medical undergraduates), as well as for surgical residents^5^.

Medical undergraduates first encounter associated queries on surgery and witness the role of surgeons during their initial clerkship years^4,9^. During the transition from being medical students to becoming medical practitioners, the learners are mostly concerned about the long hours of surgery, which may impede their enthusiasm for intraoperative learning due to reduced attention and fatigue. Moreover, offering junior learners with preparatory information or learning objectives (e.g., associated surgical anatomy or indication) before the encounter could enhance the efficiency of intraoperative teaching^10^. In contrast to the drawback of protracted and complex surgical procedures, totally implantable venous access device placement is a relatively easy surgical procedure for establishing long-term venous access for chemotherapy infusion or nutrition supplementation. The relative simplicity and short operative time enable junior surgical residents to become familiar with and even independently accomplish the entire surgical procedure^11–13^.

Most of the literature on intraoperative teaching has focused on the conceptual level, with little data on the practicable teaching process, especially for medical undergraduates^2,14^. Moreover, self-directed learning via mobile phones or tablets is currently being widely applied via modern transmission technologies, thereby presenting a suitable and practicable teaching modality for intraoperative teaching^15,16^. This study focused on learning effectiveness and perceptions to clarify the effects and feasibility of intraoperative teaching among first-year medical clerks through the modified BID model during implantable infusion port placement. Moreover, the outcomes of the objective assessment for practicing simple interrupted suturing skills were concomitantly evaluated.

## Materials and methods

**Figure 1:**
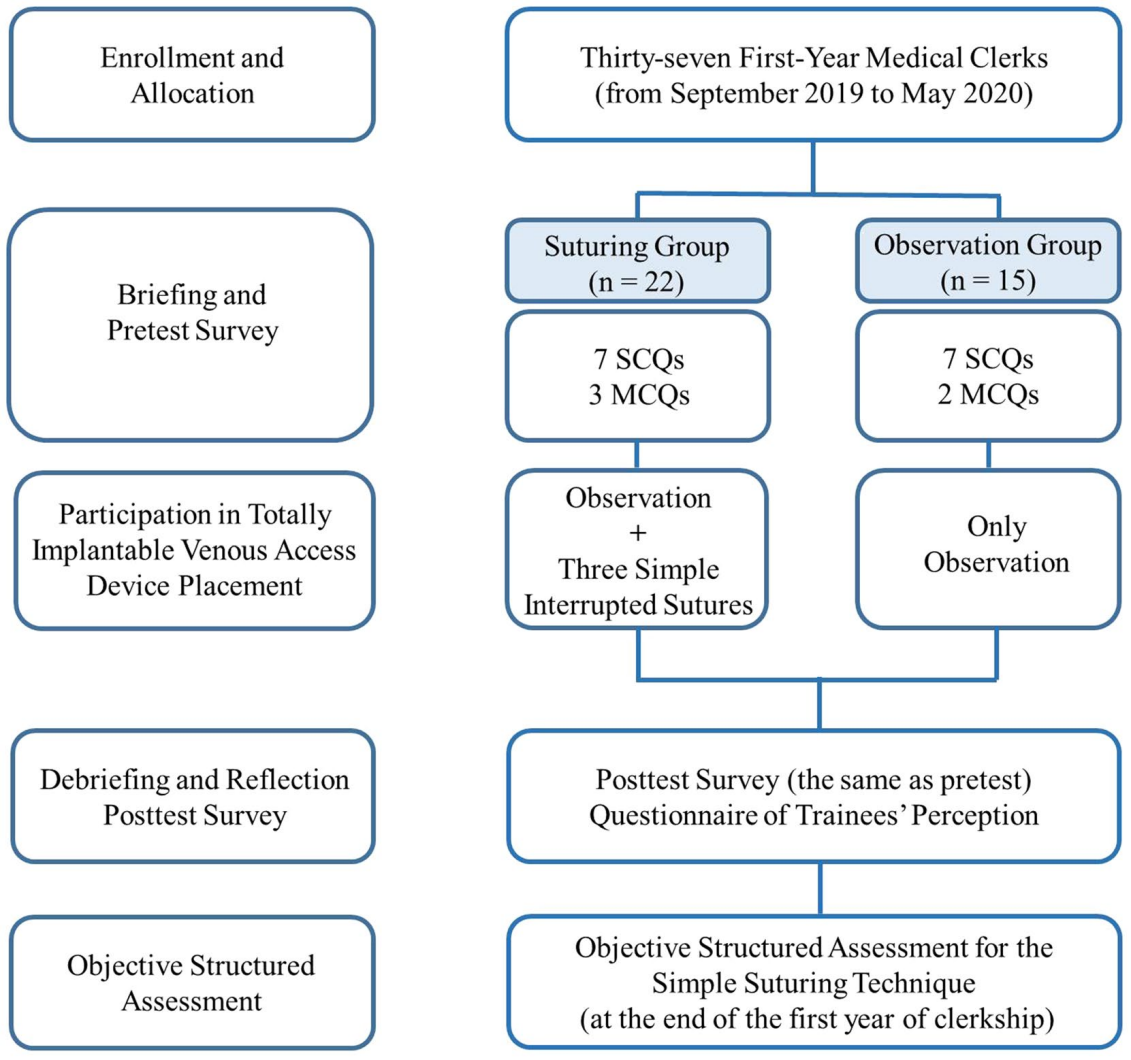
Flow diagram of the study design. *MCQ* multiple-choice question, *SCQ* single-choice question.

This prospective, non-randomized trial was performed at the Department of Surgery, Kaohsiung Medical University Hospital, Taiwan (the affiliated hospital of the Kaohsiung Medical University School of Medicine) and was conducted from September 2019 to May 2020 after approval from the Institutional Review Board of Kaohsiung Medical University Hospital (KMUHIRB-E[II]-20200049). The participants were 37 first-year medical clerks without previous suturing experience and working their shift at the Division of Thoracic Surgery. All participants were thoroughly informed of the study design and protocols before participation, and they were divided into two groups: suturing group (the intervention group with suturing practice intraoperatively) and observation group (the control group without suturing practice intraoperatively). Although the observation group had no intraoperative suturing practice, they received the similar, standard lectures and underwent suturing practice on the artificial models before the final assessment. Informed consent was obtained from all individual participants included in the study. All procedures performed in studies involving human participants were in accordance with the ethical standards of the institutional and/or national research committee and with the 1964 Helsinki declaration and its later amendments or comparable ethical standards. The datasets used and/or analyzed during the current study available from the corresponding author on reasonable request. The entire study design is shown in Fig. 1.

### Modified BID model in use

#### Briefing

Instead of the original model, which employed a short, 3–5 min interaction at the scrub sink before surgery, all participants were requested to complete the pretest survey via mobile phones or tablets, which enabled the trainees to understand the learning objectives (indications, anesthetic methods, venous access, and associated surgical anatomy) before scrubbing for implantable infusion port placement^4^. The pretest survey would not only establish the understanding of the learning objectives, but would also guide the surgical educator about the content of intraoperative teaching and ensure its quality.

The pretest survey consisted of seven single-choice questions (including indication, anesthetic method, venous access, surface landmark identification, catheter tip location, associated suture material for port fixation, and wound closure) and two multiple-choice questions (including identifying the surgical anatomy and simple suturing for portal anchoring/wound approximation) (Table 1 and Supplementary eTable 1). The third multiple-choice question, which consisted of a seven-point scoring checklist for assessing the performance of simple interrupted suturing, was designed as a pretest survey during the midterm of the entire study and was completed by 22 participants (suturing group) (Table 2). All these surveys were developed by the authors (CSL and PCC) and were administered in a close-ended format, enabling the participants to accomplish the survey easily and quickly via smartphones or tablets.

**Table 1:** Results of the pre-/posttest survey of totally implantable venous access device placement (Single-Choice Questions).

	All (n = 37)			Observation group (n = 15)		Suturing group (n = 22)			Inter-compared posttest	
	Pretest (%)	Posttest (%)	*t*-test (*p*-value)	Pretest (%)	Posttest (%)	*t*-test (*p*-value)	Pretest (%)	Posttest (%)	t-test (p-value)	*t*-test (*p*-value)
Indication										
Correct (chemotherapy)	100.00	100.00	N/A	100.00	100.00	N/A	100.00	100.00	N/A	N/A
Incorrect (long-term nutrition with difficult venous access)	0.00	0.00		0.00	0.00		0.00	0.00		
Method of anesthesia										
Correct (laryngeal mask anesthesia)	81.08	78.38	0.6609	66.67	60.00	0.5816	90.91	90.91	1.0000	0.0440*
Incorrect (intravenous general anesthesia or endotracheal general anesthesia)	18.92	21.62		33.33	40.00		9.09	9.09		
Surface landmark identification										
Correct (infra-clavicular region)	94.59	94.59	1.0000	86.67	100.00	0.1643	100.00	90.91	0.1621	0.1621
Incorrect (neck)	5.41	5.41		13.33	0.00		0.00	9.09		
Venous access										
Correct (cephalic vein)	29.73	70.27	< 0.0001**	46.67	73.33	0.0281*	18.18	72.73	0.0003**	0.7482
Incorrect (subclavian vein or internal jugular vein/venipuncture)	70.27	29.73		53.33	26.67		81.82	27.27		
Location of catheter tip										
Correct (below carina)	16.22	64.86	0.0001**	26.67	53.33	0.2711	13.64	68.18	0.0001**	0.4269
Incorrect (above carina or superior vena cava)	83.78	35.14		73.33	46.67		86.36	31.82		
Suture material for portal fixation										
Correct (VICRYL^®^)	24.32	61.54	0.0018**	13.33	40.00	0.0824	27.27	72.73	0.0079**	0.1064
Incorrect (ETHILON^®^ or PROLENE^®^)	75.68	38.46		86.67	60.00		72.73	27.27		
Appropriate suture material for fascia/skin closure										
Correct match (Fascia -VICRYL^®^; skin–ETHILON^®^)	72.97	91.89	0.1098	53.33	80.00	0.3840	86.36	100.00	0.0829	0.0961
Incorrect matches	27.03	8.11		46.67	20.00		13.64	0.00		

*p < 0.05, **p < 0.01.

*N/A* not applicable.

**Table 2:** Results of the 7-point scoring checklist for assessing performance of simple interrupted suturing (suturing group) (n = 22) (multiple-choice question No. 3).

	Pretest (%)	Posttest (%)	*t*-test (*p*-value)
Which steps were essential to simple interrupted suturing with ETHILON^®^?			
Held at 1/2–1/3 from the tip	90.91	100.00	0.1621
Angle = 90° ± 20°	90.91	100.00	0.1621
Removing the needle along the curve	90.91	100.00	0.1621
Using pulley concept or walking along the suture	86.36	100.00	0.0829
Correct C loop, complete 1st knot	90.91	100.00	0.1621
Inverse loop, complete 2nd knot	77.27	95.45	0.0425*
All knots laid on the side (not over the incision)	81.82	90.91	0.3287

*p < 0.05.

### Totally implantable venous access device placement and associated intraoperative teaching

All Year 1 medical clerks scrubbed in and participated as the first assistant throughout the surgery after finishing the pretest survey. Only one or two first-year medical clerks were allowed for each implantable infusion port placement to maintain the quality of intraoperative teaching. The patient was placed in a supine position after anesthesia induction (laryngeal mask anesthesia). The right infraclavicular region or the right neck is usually chosen as the target surgical site for implantable infusion port placement. The associated surgical anatomy was identified and introduced during the surgery. All surgeries were performed via venotomy by a single senior surgical consultant (PCC). A portable chest X-ray was used to confirm the exact location of the catheter tips after intraoperative catheter indwelling into the cephalic vein. The catheter was routinely connected to the catheter connector of the portal, and suturing was performed using Coated VICRYL^®^ 3–0 (ETHICON Inc., Cincinnati, OH, USA) for portal anchoring to the muscle/fascia approximation and ETHILON^®^ 4–0 (ETHICON Inc.) for superficial wound closure. Those who took the pretest survey containing the 7-point scoring checklist for assessing the performance of simple interrupted suturing (suturing group) were asked to perform three simple interrupted sutures for superficial wound closure using ETHILON^®^ 4–0 under the supervision of a senior surgical consultant (PCC). Those who only observed throughout the entire procedure were designated as the observation group.

#### Debriefing

The trainees were allowed to reflect on their viewpoints or queries about their hands-on practice and the surgery based on the spirit of “rules, reinforcement, and correction” after the completion of implantable infusion port placement^4^. Moreover, all the trainees had to finish the posttest survey (identical to the pretest survey) via mobile phones/tablets to instill and retain the learning objectives of the surgery.

### The trainee’s perceptions of intraoperative teaching

Aside from enhancing the learning objectives of the surgery via the pre-/posttest survey, all participants were requested to complete the questionnaire on the trainee’s perceptions of intraoperative teaching (Mandarin version, designed by CSL), which consisted of 14 quantitative evaluations of their participation during totally implantable venous access device placement with a Cronbach’s alpha = 0.909, indicating a satisfactory internal consistency reliability for all composite scales, and an acceptable content validity of 0.681 (Supplementary eTable 2). Exploratory factor analysis identified three factors with a total variance explained of 81.398%. The items of this questionnaire were based on the five-point Likert scale (strongly disagree, 1 point; disagree, 2 points; neither disagree nor agree, 3 points; agree, 4 points; and strongly agree, 5 points). Within this questionnaire, the medical undergraduates were prompted to provide feedback on the different sections of the training (before surgery, intraoperatively, and after surgery). The survey questions are presented in Supplementary eTable 2, including the four domains of perception toward the environment of intraoperative teaching and the interaction between the surgical educator and learners before, during, and after the surgery.

### Objective structured assessment for simple interrupted suturing

All trainees had to undergo an objective structured assessment of their performance of simple interrupted suturing, which is the end part of the formal curriculum for first-year clerkship (May 2020). The medical undergraduates had to follow the same instructions for similar simple interrupted suturing within 8 min under aseptic conditions, including six domains on the simple interrupted suturing technique (ETHILON^®^ suture, using instruments correctly, proper entry and exit of sutures, knot tying, wound care, and safe disposal of sharp objects), with 2 points for each domain and a maximum of 12 points (0–12 points). The students’ performance of simple interrupted suturing was evaluated by certified senior surgeons.

### Data analysis

The primary outcome of this study was the trainee’s perceptions of intraoperative teaching; the secondary outcome was the results of pre- and posttest surveys and the questionnaires and the participants’ performance of the objective structured assessment for simple interrupted suturing. All statistical tests were conducted using SPSS for Windows version 19.0 (released 2010; IBM Corp., Armonk, NY, USA). The results of the pre-/posttest surveys and the questionnaire on intraoperative teaching were stored and analyzed on Microsoft^®^ Excel (Microsoft Corp., Redmond, WA, USA). Full details are provided in Tables 1, 2, 3 and 4 and Supplementary eTable 1. Descriptive statistics were used to summarize the demographic and sex ratio data. The differences in teaching interactions were analyzed at different stages (before, during, and after the surgery) and in both groups (suturing group vs. observation group). All data are presented as mean ± standard deviation. Associated correlations were determined using a two‐tailed paired *t*-test. A *p*-value less than 0.05 was considered statistically significant.

**Table 3:** Results of the pre-/posttest survey during totally implantable venous access device placement (n = 37).

	All (n = 37)			Observation Group (n = 15)			Suturing Group (n = 22)		
	Pretest (Average)	Posttest (Average)	*t*-test (*p*-value)	Pretest (Average)	Posttest (Average)	*t*-test (*p*-value)	Pretest (Average)	Posttest (Average)	*t*-test (*p*-value)
MCQ1: assessing surgical anatomy identification (8 points)	4.65	6.49	< 0.0001**	4.53	6.13	0.0077**	4.73	6.73	0.0034**
MCQ 2: assessing suturing for portal anchoring/wound approximation (3 points)	2.19	2.70	0.0032**	2.13	2.73	0.0140*	2.23	2.68	0.0664
MCQ 3: assessing performance of simple interrupted suturing task (7 points)	N/A	N/A	N/A	N/A	N/A	N/A	6.09	6.86	0.0501

*p < 0.05, **p < 0.01.

*MCQ* multiple-choice question, *N/A* not applicable.

**Table 4:** Questionnaire on trainee’s perceptions toward intraoperative teaching (14 quantitative questions).

	All (n = 37)		Observation group (n = 15)	Suturing group (n = 22)	*t*-test (*p*-value)
	Average points (1–5 points)	Standard deviation	Average points (1–5 points)	Average points (1–5 points)	
Environment in operating room					
Q1. In the operating room, the educator will contribute more time and energy to teach me	4.57	0.603	4.27	4.77	0.0128*
Before surgery					
Q2. The surgical educator will discuss with me actively before the surgery	4.30	0.878	3.80	4.64	0.0047**
Q3. I will discuss with the surgical educator actively before the surgery	4.00	0.882	3.47	4.36	0.0022**
Q4. I will observe before surgery	4.22	0.672	3.93	4.41	0.0295*
Q5. I’m satisfied with the educator’s teaching content before the surgery	4.30	0.812	3.87	4.59	0.0130*
During surgery					
Q6. The educator will conduct teaching related to the surgery intraoperatively	4.54	0.900	4.20	4.77	0.0832
Q7. The educator will communicate with me during the surgery, instead of aiming to keep the surgery short	2.43	1.444	1.73	2.91	0.0059**
Q8. The educator will communicate with me during the surgery to enable the surgery to proceed smoothly while allowing me to learn something	4.54	0.605	4.40	4.64	0.2584
Q9. I will raise questions appropriately during the surgery	4.35	0.633	4.00	4.59	0.0068**
Q10. I’m satisfied with the educator’s teaching during the surgery	4.46	0.836	4.20	4.64	0.1426
After surgery					
Q11. The educator will provide immediate feedback based on my strong points during the surgery	4.16	0.898	3.80	4.41	0.0498*
Q12. The educator will immediately provide suggestions based on my shortcomings during the surgery	4.41	0.762	4.07	4.64	0.0234*
Q13. I will discuss with the educator actively about my performance today	3.68	1.002	3.13	4.05	0.0054**
Q14. I’m satisfied with the educator’s feedback after the surgery	4.54	0.767	4.33	4.68	0.1733

*p < 0.05, **p < 0.01.

## Results

This study enrolled 22 male and 15 female clerks who attended 20 totally implantable venous access device placements under supervision. Among these participants, 27 (73%) were from the School of Medicine, while the remaining 10 (27%) were from the School of Postbaccalaureate Medicine. No postprocedural complications (wound infection, seroma, hematoma, or wound dehiscence) on the 30th postoperative day or needlestick injuries were observed during any of the surgeries. The response rates for the pre- and posttest surveys were both 100%. Most of the participants completed the posttest survey and the questionnaire on intraoperative teaching within 1 week after participating in the surgery (81.1%), and all participants completed the posttest survey within 1 month.

### Results of single-choice questions

All participants had to answer seven single-choice questions to retain the definite learning objectives during the surgery for the pre- and posttest surveys. Most trainees could identify without difficulty the related indication, anesthetic method, surgical site, and appropriate suture for the fascia/skin closure before their hands-on activity. After the surgery, trainees could reconfirm the precise venous access, catheter tip location, and suture materials for portal fixation (*p* < 0.05). Compared with the observation group, the suturing group identified the correct anesthetic method (laryngeal mask anesthesia) for the surgery (*p* < 0.05) (Table 1).

### Results of multiple-choice questions

All participants had to complete two multiple-choice questions (No. 1 and 2) in the pre- and posttest surveys to enhance intraoperative learning during the surgery (Supplementary eTable 1). Most of the participants showed improved recognition of related surgical anatomic structures (6.49 vs. 4.65, *p* < 0.0001) and suturing for portal anchoring and wound approximation (2.70 vs. 2.19, *p* < 0.05) (Table 3). The 22 participants in the suturing group who performed simple interrupted suturing for superficial wound closure under supervision at the end of the placement took another pre- and posttest survey (multiple-choice question No. 3) to assess key elements in practicing simple interrupted suturing (Table 2). The total scores for No. 3 multiple-choice question did not reach statistical significance despite hands-on experience (6.86 vs. 6.09, *p* = 0.0501) (Table 3).

### Outcomes of the questionnaire on the trainee’s perceptions of intraoperative teaching

In addition to augmenting the learning objectives via pre- and posttest surveys of the trainees’ participation, exploring the perceptions of intraoperative teaching was another important issue. Based on the current questionnaire (Supplementary eTable 2), a better perception of intraoperative teaching was reported by participants in suturing group than in observation group, especially for “contributing more time and energy in the operating room,” “more discussion between the educator and trainees before surgery,” “more observation opportunities for trainees prior to performing suturing,” “more satisfaction with teaching content before surgery,” “more communication without hesitation during surgery,” “appropriately raising questions during surgery,” “more immediate feedback on trainees’ strong points/shortcomings after surgery,” and “actively discussing trainees’ performance after participation” (Table 4).

### Outcomes of the objective structured assessment for simple interrupted suturing

All medical undergraduates had to complete an objective structured assessment for the simple suturing technique scheduled at the end of first year of clerkship (May 2020), including the participants in this study. The difference in scores on the objective structured assessment between suturing group and observation group was not statistically significant (8.7 ± 1.8 vs. 7.2 ± 3.7; *p* = 0.104).

## Discussion

Intraoperative teaching is the cornerstone of the development of surgical skills. The present study sought to determine the feasibility of the modified BID model as an adjunct for matching intraoperative teaching and learning between a single senior surgical educator and first-year medical clerks in an affiliated teaching hospital of a medical university. This prospective, non-randomized study found that the modified BID model was a practicable solution for surgical educators to convey the essential learning objectives (e.g., surgical indication, anesthetic method, and associated surgical anatomy), as well as the key elements of simple suturing practice, via the modern technology of webinars via mobile phones before/after totally implantable venous access device placement efficiently. Moreover, the junior learners could obtain a better perception of intraoperative teaching via the hands-on experience of practicing simple interrupted suturing.

In medical education, evaluating previous achievements in teaching and learning is difficult, especially in intraoperative teaching, an environment full of uncertainty and unorganized teaching material due to the reality of unpredictable clinical situations, shortage of human resources, and emphasis on operating room efficiencies^17^. Intraoperative teaching with immediate feedback is of critical importance, and introducing a structured model in place of traditional observership is essential in modern surgical education to enhance the learning experience (perception and efficiency) of medical undergraduates in the operating room. The BID model not only meets the clinical needs of junior learners, but also enhances the teaching efficiency and performance of surgical educators^5–8^. The original BID model was initiated by Roberts et al. in 2009 to assist surgical educators in clarifying the learning objectives for the learners before surgery, establish bidirectional communication during the surgery, and then reflect on the goals achieved and how learners can improve themselves clinically going forward^5^.

Based on the core spirit of “briefing, intraoperative teaching, and debriefing” via mutual and verbal communication, the pre- and posttest surveys (single- and multiple-choice questions) were also introduced via mobile learning, which could precisely transmit key learning objectives and consolidate these domains after participation^15,16^. As well as enabling immediate, specific feedback to help learners understand mistakes or doubts, the BID model could achieve higher educational goals by concomitantly mitigating the uncertainty during intraoperative teaching. After their participation, the junior learners in the current study recognized the details of the surgery, including selecting proper venous access, confirming the catheter tip location, and choosing the suture materials for portal fixation (Table 1). This model could also help improve the learners’ focus throughout the surgery.

Fatigue, long hours, and potential physical hardships are key concerns of medical undergraduates at the beginning of surgical clerkships. Thus, establishing an efficient model of intraoperative teaching by participating in a relatively short surgical procedure is necessary to maintain the trainees’ attention^4^. Totally implantable venous access device placement, a relatively easy and short surgical procedure, is commonly performed by surgical residents under direct supervision or independently^11–13^. In the present study, venotomy via the cephalic vein was directly adopted to accomplish catheter placement other than direct venipuncture. Given the fact that the surgical site was superficialized to better visualize the surgical anatomy, the difficulty and domains of interest of medical undergraduates during their surgical clerkships, the learners could better identify the surrounding anatomic structures clearly during their participation^4,18^. Furthermore, with the simplicity and limited surgical manipulation timeframe of totally implantable venous access device placement, it allowed junior learners to thoroughly concentrate on the entire procedure and accordingly enhance learning efficiency.

Compared with other disciplines of medicine, surgery has a strong emphasis on hands-on practical training^1,19^. Moreover, suturing is a fundamental skill and is a core competency for medical undergraduates^20^. In the present study, first-year medical clerks in the suturing group were allowed to perform three simple interrupted sutures at the end of the surgery under supervision. These junior learners received immediate, critical corrections from the surgical educator after each practice, which improved the learners’ perception toward intraoperative teaching compared with observation group, including increased contributions from the surgical educator, more discussions with the educator, more demonstrations that consolidated the learning objectives before actual performing actual suturing, absence of hesitation in discussing with the surgical educator during surgery, more immediate feedback from the educator, and active discussion of performance. Thus, the trainee’s autonomy is augmented, and their motivation is stimulated through the hands-on experience^21^.

The clinical significance of the hands-on experience could not be overemphasized, given the increasing active roles of medical undergraduates in the operating room and the fundamental requirements in surgical clerkship^20–22^. Concerns on perisurgical safety remain, although many studies have favored hands-on practice for intraoperative teaching^21–24^. The use of pretest surveys (multiple-choice question No. 1–3) via mobile learning was introduced, which enabled the participants to become familiar with the associated surgical anatomy in totally implantable venous access device placement and the key elements in practicing simple interrupted suturing to reduce and even prevent possible adverse events (e.g., wound complications or needlestick injuries) related to hands-on suturing practice by junior learners. In suturing group, better recognition of surgical anatomy was observed (Tables 2 and 3; Supplementary eTable 1), and no direct wound complications on the 30th postoperative day or needlestick injuries were reported during the follow-up. We believe that the modified BID model could help trainees acquire fundamental knowledge on minor surgeries, such as totally implantable venous access device placement, without causing adverse postoperative outcomes due to hands-on suturing practice.

This study has some limitations. First, the current study was limited by the relatively small sample size (n = 37) and short timeline, which may hinder the generalizability of the results. Future expansion of the entire investigation is necessary to validate these results and draw a firm conclusion. Second, although positive perceptions toward intraoperative teaching were statistically significant in suturing group, the drawbacks of a non-randomized study design and mutual expectations between the surgical educator and learners may not provide an accurate measurement of the trainees’ actual perceptions. Third, only a single senior surgical educator with only one training unit was recruited, and the favorable conclusion obtained in this study may not be reflective of all surgical educators. Fourth, although the scores on the objective structured assessment of simple interrupted suturing were relatively higher in suturing group, they were not statistically significant (8.7 ± 1.8 vs. 7.2 ± 3.7; *p* = 0.104). Poor skill retention due to the transition in the trainees’ clerkships with different time intervals between initial intraoperative teaching and subsequent objective structured assessment for simple suturing technique (2–32 weeks) may explain this outcome.

## Conclusion

Our study demonstrated the clinical significance of the modified BID model as a useful adjunct of intraoperative teaching for the surgical educator and first-year medical clerks in a university-based teaching hospital. Furthermore, the modified BID model with hands-on practice can match intraoperative teaching and learning via the augmentation of good learning perceptions for medical undergraduates’ participation in totally implantable venous access device placement.

### Supplementary Information

Supplementary Tables.

Supplementary Tables.

## Data Availability

The datasets used and/or analyzed during the current study available from the corresponding author on reasonable request.
