# Supplementary material for: Matching intraoperative teaching and learning for medical undergraduates via modified briefing-intraoperative teaching-debriefing (BID) model
Source: Sci Rep. 2023 Aug 23;13:13732. doi: 10.1038/s41598-023-40755-9 (PMC10447542; doi:10.1038/s41598-023-40755-9)
Supplement: Supplementary file 1 — Supplementary Tables. [file 41598_2023_40755_MOESM1_ESM.docx]

**Supplementary Information**

**Title:** Matching Intraoperative Teaching and Learning for Medical Undergraduates via Modified Briefing-Intraoperative Teaching-Debriefing (BID) Model

**Authors:** Yu-Tang Chang^1,2^, Nan-Chieh Chen^3^, Shu-Hung Huang^2,4,5^, Chung-Sheng Lai^2,4^, Cheng-Sheng Chen^2,6^, Ting-Wei Chang^7^, Po-Chih Chang^7,8,9,10*^

**eTable 1. The results of pre-/posttest survey during totally implantable venous access device placement (multi-choice question)**

|  | **All (n = 37)** | | | **Observation Group (n = 15)** | | | **Suturing Group (n = 22)** | | |
| --- | --- | --- | --- | --- | --- | --- | --- | --- | --- |
|  | **Pretest**  **(%)** | **Posttest**  **(%)** | ***t-test***  ***(p-value)*** | **Pretest**  **(%)** | **Posttest**  **(%)** | ***t-test***  ***(p-value)*** | **Pretest**  **(%)** | **Posttest**  **(%)** | ***t-test***  ***(p-value)*** |
| **MCQ 1: Surgical anatomy identification** |  |  |  |  |  |  |  |  |  |
| Epidermis | 54.05 | 75.68 | 0.0094^**^ | 53.33 | 73.33 | 0.0824 | 54.55 | 77.27 | 0.0566 |
| Subcutaneous layer | 62.16 | 78.38 | 0.1098 | 73.33 | 73.33 | 1.0000 | 54.55 | 81.82 | 0.0555 |
| Fascia layer | 54.05 | 72.97 | 0.0508 | 46.67 | 66.67 | 0.2711 | 59.09 | 77.27 | 0.1035 |
| Deltoid muscle | 45.95 | 86.49 | <0.0001^**^ | 33.33 | 73.33 | 0.0086^**^ | 54.55 | 95.45 | 0.0010^**^ |
| Pectoralis major muscle | 56.76 | 89.19 | 0.0002^**^ | 46.67 | 80.00 | 0.0192^*^ | 63.64 | 90.45 | 0.0051^**^ |
| Cephalic vein | 67.57 | 83.78 | 0.0831 | 60.00 | 86.67 | 0.0406^*^ | 72.73 | 81.82 | 0.4923 |
| Deltoid-pectoral groove | 48.65 | 75.68 | 0.0008^**^ | 60.00 | 73.33 | 0.1643 | 40.91 | 77.27 | 0.0023^**^ |
| Clavicle | 75.68 | 86.49 | 0.1601 | 80.00 | 86.67 | 0.5816 | 72.73 | 86.36 | 0.1858 |
| **MCQ 2: Suturing for portal anchoring/wound approximation** |  |  |  |  |  |  |  |  |  |
| portal anchoring | 83.78 | 91.89 | 0.2625 | 86.67 | 100.00 | 0.1643 | 81.82 | 86.36 | 0.6652 |
| fascia layer closure | 59.46 | 81.08 | 0.0187^*^ | 53.33 | 80.00 | 0.0406^*^ | 63.64 | 81.82 | 0.1621 |
| skin closure | 75.68 | 97.30 | 0.0033^**^ | 73.33 | 93.33 | 0.0824 | 77.27 | 100.00 | 0.0215^*^ |

^*^:*p* < 0.05 ^; **^: *p* < 0.01. MCQ: multiple-choice question

**eTable 2. Questionnaire of trainee’s perceptions towards intraoperative teaching during totally implantable venous access device placement**

|  | **1**  (strongly disagree) | **2**  (disagree) | **3**  (neutral) | **4**  (agree) | **5**  (strongly agree) |
| --- | --- | --- | --- | --- | --- |
| **Environment of Intraoperative Teaching** | | | | | |
| Q1. In the operating room, the educator will contribute more time and energy to teach me. |  |  |  |  |  |
| **Before Surgery** | | | | | |
| Q2. The surgical educator will discuss with me actively before the surgery. |  |  |  |  |  |
| Q3. I will discuss with the surgical educator actively before the surgery. |  |  |  |  |  |
| Q4. I will preview before the surgery. |  |  |  |  |  |
| Q5. I'm satisfied with the educator's teaching content before the surgery. |  |  |  |  |  |
| **During Surgery** | | | | | |
| Q6. The educator will conduct teaching related to the surgery intraoperatively. |  |  |  |  |  |
| Q7. The educator will communicate with me during the surgery, instead just to let the surgery be ended earlier. |  |  |  |  |  |
| Q8. The educator will communicate with me during the surgery, that would let the surgery proceed smoothly and I can learn something concomitantly. |  |  |  |  |  |
| Q9. I will raise questions appropriately during the surgery. |  |  |  |  |  |
| Q10. I'm satisfied with the educator's teaching during the surgery. |  |  |  |  |  |
| **After Surgery** | | | | | |
| Q11. The educator will provide immediate feedback based on my strong points during the surgery. |  |  |  |  |  |
| Q12. The educator will provide immediate suggestion based on my shortcomings during the surgery.” |  |  |  |  |  |
| Q13. I will discuss with the educator actively about my performance today. |  |  |  |  |  |
| Q14. I'm satisfied with the educator's feedback after the surgery. |  |  |  |  |  |
